# Stratification of insect diversity and daily activity patterns in the West African virgin forest Taï assessed by entomological Lidar

**DOI:** 10.1038/s41598-025-05200-z

**Published:** 2025-07-15

**Authors:** Hampus Månefjord, Assoumou Saint-Doria Yamoa, Yatana Adolphe Gbogbo, Lauro Müller, Anna Runemark, Benoit Kouassi Kouakou, Rabbi Boateng, Andrew Atiogbe Huzortey, Isaac Kwame Badu, Niklas Wahlberg, Mikkel Brydegaard, Jérémie T. Zoueu, Benjamin Anderson, Meng Li

**Affiliations:** 1 Department of Physics, Lund University, Sölvegatan 14C, 22363 Lund, Sweden; 2 Institut National Polytechnique Félix Houphouët-Boigny de Yamoussoukro, Yamoussoukro, Côte d’Ivoire; 3 Department of Biology, Lund University, Sölvegatan 35, 22362 Lund, Sweden; 4 Department of Physics, University of San-Pedro, BP 1800, San-Pedro, Côte d’Ivoire; 5 Laser and Fibre Optics Centre (LAFOC), Univeristy of Cape Coast (UCC), Cape Coast, Ghana; 6 Department of Conservation Biology and Entomology, University of Cape Coast (UCC), Cape Coast, Ghana; 7 Biological Museum, Department of Biology, Lund University, Sölvegatan 37, 22362 Lund, Sweden; 8 Norsk Elektro Optikk, Østensjøveien 34, 0667 Oslo, Norway

## Abstract

Lidar technology was utilized to non-destructively explore the diverse insect life in the Taï virgin forest of Côte d’Ivoire. This study employed an entomological Lidar scanned at different elevation angles, combined with traditional insect trapping at different canopy heights, to investigate the composition and spatial–temporal distribution of insects within the forest canopy. We detected stratified patterns of insect activity at various canopy heights, revealing differences in the composition of Lidar signals reflecting divergent species composition with height and time-of-day, indicative of height-dependent insect biodiversity. The optical properties of captured insects, including wing specularity and polarimetric response, were analyzed and correlated with Lidar signals, revealing distinct insect clusters and activity patterns at different canopy heights. These findings show the potential of using Lidar to non-invasively assess insect diversity continuously across complex canopies to uncover species composition in ecosystems with high species diversity.

**Supplementary Information:**

The online version contains supplementary material available at 10.1038/s41598-025-05200-z.

## Introduction

Tropical virgin forests stand as some of the world’s richest reservoirs of biodiversity, yet they also rank among the most threatened biomes. To conserve the species richness in these regions, it is crucial to understand the distribution and predictors of biodiversity^1–3^, especially for insects, which are not only the most species-rich taxa but also among the most threatened^4–6^. Recent reports have highlighted alarming declines in insect assemblages and biomass, with estimates suggesting that terrestrial insects are declining at an average rate of 11% per decade globally^7,8^. Despite their significance, our understanding of insect populations in tropical rainforests, in particular in sub-Saharan Africa, lags behind that of regions like Europe and North America^9,10^. One challenge in identifying the habitats that are key to maintaining insect diversity is the non-uniform distribution of insects within tropical canopies, making vertical stratification a key aspect to assess^11,12^. Understanding and preserving insect biodiversity in tropical forests is paramount for avoiding insect mass extinction.

Conventional methods for insect monitoring, including malaise- and baited traps^13^ and tree-branch beating^14^ offer limited spatial and temporal resolution . These methods also have inherent biases^15–17^ and are weather-dependent species composition in catches^18^. The employment, operation, and taxonomical^19^ or DNA^20^ based identification of catches of trap- and tree-branch beat catches are both time-consuming and costly and require skill and equipment that is not accessible to all. Additionally, these methods often require sacrificing captured insects, which might pose ethical concerns, especially in areas harboring endangered species^21^.

The limitations of conventional methods call for innovative, non-invasive approaches to monitor insect diversity within complex ecosystems. High spatial and temporal resolution with a retained ability to differentiate species is key for capturing true diversity. While techniques such as image- and sound recognition^22–24^ and digital holography^25^ show great potential, their field of view is often limited to a few square meters or less. Radars show promise for monitoring high-altitude insect migration on a large scale^26–29^ but can generally not be operated within forest canopies due to Radar side lobes resulting in ground clutter noise^30^. Moreover, their capacity to distinguish between species is often restricted^31^.

In this study, we address the challenge of monitoring insects in stratified environments and representing the full composition of species that are active over the day by employing entomological Lidar. We have previously demonstrated the potential of Lidar to provide detailed information on insect abundance^32,33^, distribution^34^, flight characteristics^35–37^, species richness^38,39^, powder-tagged monitoring^40,41^, and features such as wing thickness^42^, have been previously established. We expand on this work by using a near-infrared (NIR) polarization Lidar^43^ to achieve spatial (centimeter-scale) and temporal (millisecond) resolution in mapping insect activity within a virgin rainforest canopy. This study provides insight into the intricacies of insect monitoring and diversity estimation within complex habitats such as stratified forests.

## Results

### Experimental site, Lidar setup and insect trapping

**Fig. 1:**
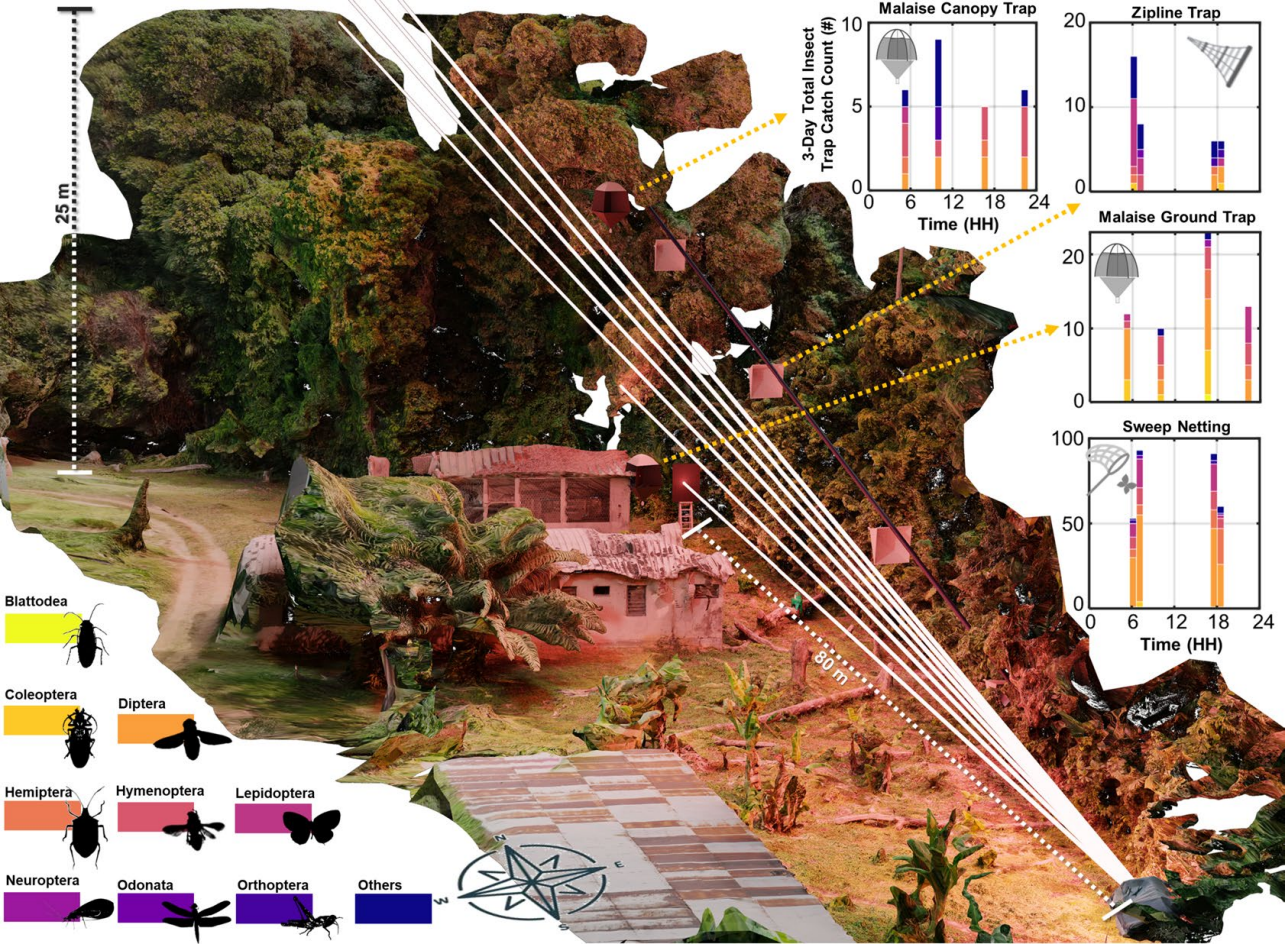
A 3D-rendered representation of the Taï virgin forest experimental site derived from 600 drone-acquired images. The texture and 3D model of the site was constructed using DroneDeploy 4.115.0 (DroneDeploy, Inc., 2022, https://www.dronedeploy.com/), and the rendering of illumination was done in Blender 3.1.0 (Blender Foundation, 2022, https://www.blender.org/). Seven white lines indicate Lidar measurements made at elevation angles ranging from 0˚ to 20˚, illustrating how the forest structure was scanned at different heights. The locations of the malaise and zipline traps are marked. Bar charts on the right show the total insect capture counts (segmented by insect orders) for each trap type over a three-day measurement period, allowing direct comparison of trapping efficacy. The 80 m ground Lidar transect was oriented to 34°N, and the canopy reached a height of 25 m at this specific site.

The study was conducted in Taï rainforest (Parc National de Taï), Côte d’Ivoire (5°49′59.5″N 7°20′32.8″W), from January 12th to 14th 2023, with measurements recorded throughout the day. Insect activity was measured through manual trapping with sweep net, Malaise traps and zipline netting and Lidar observations. The 3D-rendered area of the experimental site is illustrated in Fig. 1, marking the positions of manual trapping. The trap data shown represent cumulative insect captures spanning the full three-day measurement period. Manual trapping yielded 417 catches over the three-day measurement period, identified only down to insect order level, as higher-resolution taxonomic identification was logistically not possible, and classification to species from photographs not feasible. Our observations indicate variation in the species caught by the three different trapping methods employed; malaise at different heights, zipline, and manual sweep netting, although the catch numbers are not high enough to draw robust ecological conclusions. *Diptera* was the taxonomic group that made up the majority of catches in all traps. The zipline stood out, however, being the only trap to capture *Odonata* and *Orthoptera*, except for a few manual sweep netting catches. The temporal resolution of all traps was four times per 24-h period. Both malaise traps and the manual sweep netting resulted in higher catches during evening hours, consistent with higher levels of activity during these hours, while the zipline trap instead resulted in higher catches during morning hours.

### Spatial and temporal distribution of Lidar observations

The Lidar system recorded 19,369 insects over three days; in contrast, manual traps collected 417, a difference close to two orders of magnitude. For in-depth analysis, we included 6,962 Lidar insect observations with signal durations exceeding 25 ms. The observation included at least 80 time sample points in for co- and de-polarized signals whereby we could also estimate 80 modulation powers in logarithmically spaced frequency bins from 40 to 1666 Hz (the Nyquist frequency). This selection criterion allowed us to observe wing beat frequencies down 80 Hz.

One limitation arose from the slow-moving tripod motor, which necessitated frequent pauses for canopy scanning adjustments. As a result, the Lidar was operational for only 12% of the total measurement duration, leading to a reduced number of insect observations compared to previous studies^34,44^.

**Fig. 2:**
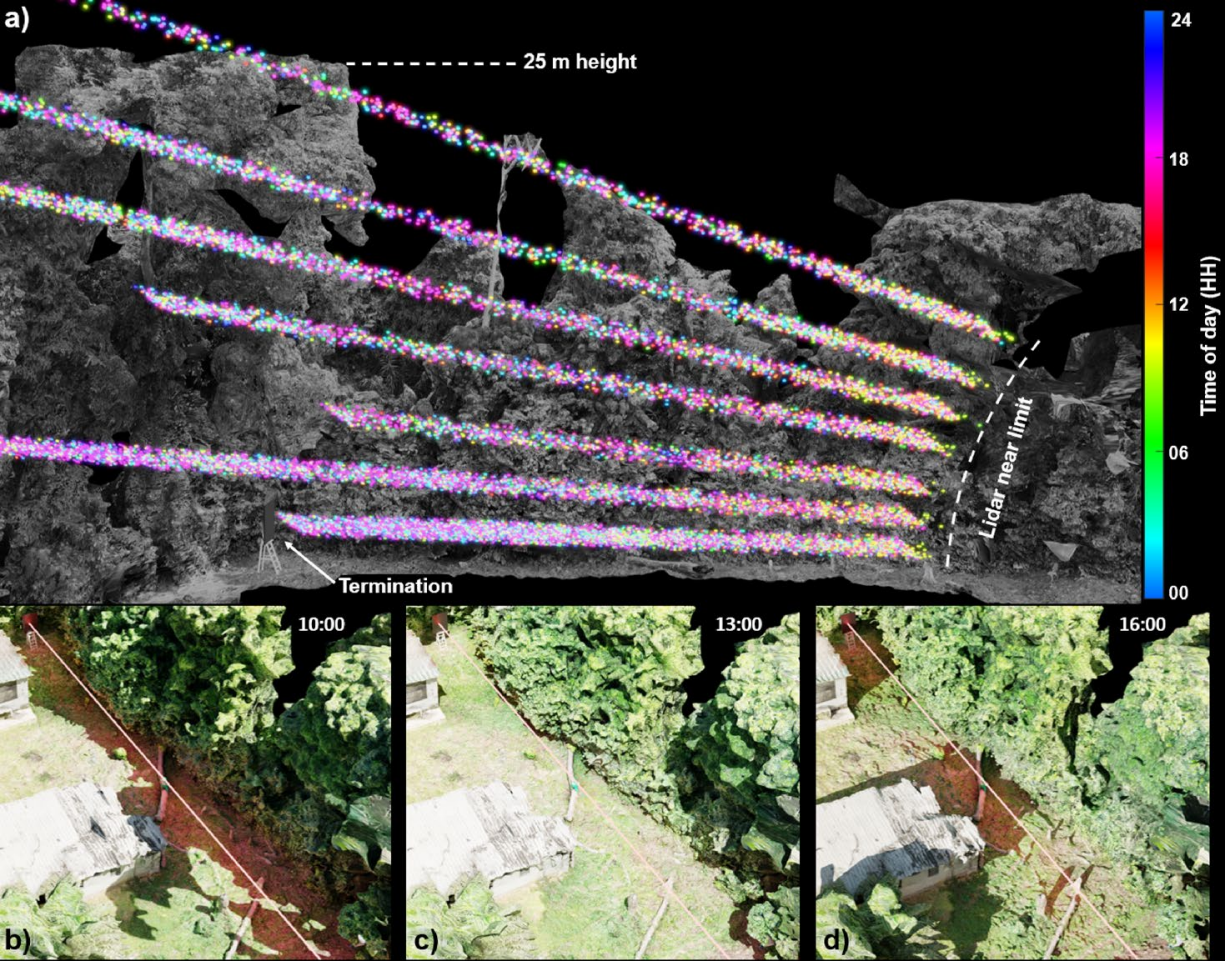
Overview of the experimental setup showcasing how insect activity varies across multiple distances and canopy levels throughout the day. (**a**) Insect scatter points (detected by the Lidar) are color-coded according to the local time of day. (**b**–**d**) Close-up, ground-level views at 10:00, 13:00, and 16:00, respectively, illustrating the laser beam’s position at elevation angle of 0° and the progression of shaded areas as the sun moves across the sky. These panels help visualize how both sunlight and shade influence insect activity at different times of day.

Sunlight exposure and canopy structure seemingly affected insect prevalence and species composition. Lidar insect signals are recorded throughout the day, illustrated with color-coding indicating the time of observation (Fig. 2a). We simulated sun positions throughout the day to correlate insect activity with shaded areas and sunlight exposure. The shaded and sunlit areas changed across the transect during the daily cycle (Fig. 2b–d). We reveal a higher insect signal density within the shade of the large canopy compared to the open area at closer range when interrogating data from elevation angles 13° and 17° in Fig. 2a. This difference in density cannot be explained by the Lidar beam’s characteristics, suggesting that factors such as direct sunlight exposure may be influencing insect distribution patterns. Additionally, closer-range observations show an increase in activity near a vegetation wall around 10 AM (Fig. 2a; indicated by yellow points). This observation adds to the evidence that many insects are active in the shade, particularly smaller insect species that might be more susceptible to desiccation from direct sunlight.

**Fig. 3:**
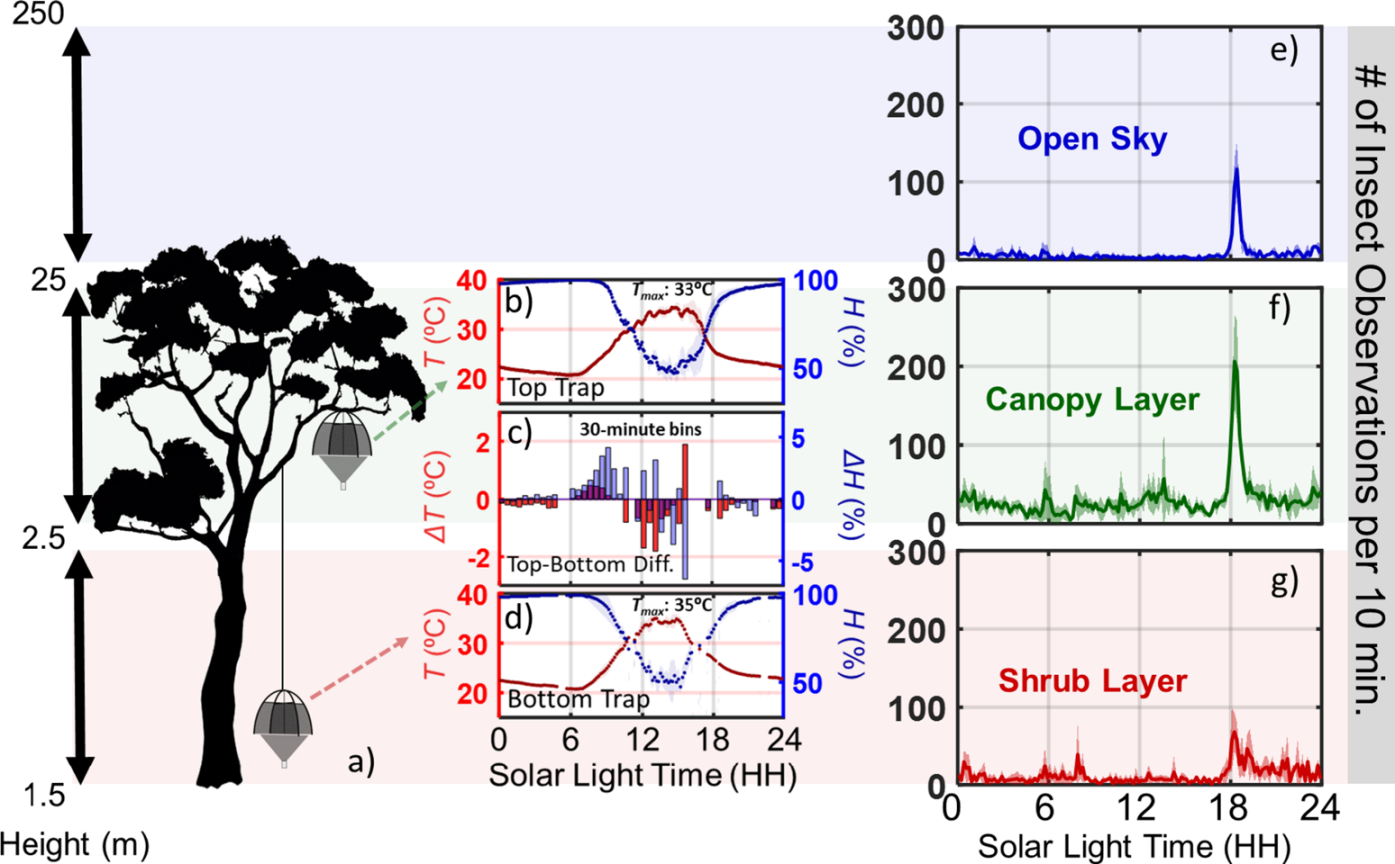
A representation of insect activity across the varied layers of the forest canopy. (**a**) The position of the Malaise traps on a tree, differentiating tree heights into the shrub layer (1.5–2.5 m), the canopy layers (2.5 m–25 m), and the open sky (25 m and above). Note that the depiction is not to scale. (**b**,**d**) show temperature (red) and relative humidity (blue) measurements from the top and bottom Malaise traps over three days. Solid lines represent the mean, while shaded regions highlight the daily variation. (**c**) Variance in temperature and humidity between the top and bottom traps (plotted in 30-min bins). (**e**–**g**) Insect activity patterns (# of observations per 10 min) at the different canopy levels, with lines showing mean values and shaded portions reflecting standard deviation variations over the 3-day measurement interval.

We documented insect activity and diversity at three heights: the Shrub layer (1.5 to 2.5 m), the Canopy layer (2.5 to 25 m), and the Open Sky layer (above 25 m) to identify variation in species composition and activity patterns across these zones. Temperature and humidity were recorded at 1 m and 20 m heights to assess environmental conditions within the Shrub and Canopy layers, revealing that the Shrub layer is ~ 2 °C warmer during peak hours (12:00–16:00), see Fig. 3. There is also a notable difference in morning humidity, with the Canopy layer having ~ 5 percentage points higher humidity than the Shrub layer. The Open Sky layer (above 25 m) exhibited a prominent activity peak between 18:00–20:00 (38% of the observations for the layer). In contrast, the Canopy layer showed both a similar evening activity peak (25%) and sustained activity throughout daylight hours (9:00–17:00, 73% of observations). There is also some nocturnal activity and an early morning surge in the Canopy layer, which thus is the layer showing the most consistent insect activity. The Shrub layer displayed an evening peak (18:00–20:00, 26% of layer observations), significant nocturnal activity (20:00–2:00, 32% of layer observations), and morning activity (05:00–9:00, 15% of layer observations), with minimal activity during warmer daylight hours (9:00–17:00).

### Optical characterization of insect specimen signals

**Fig. 4:**
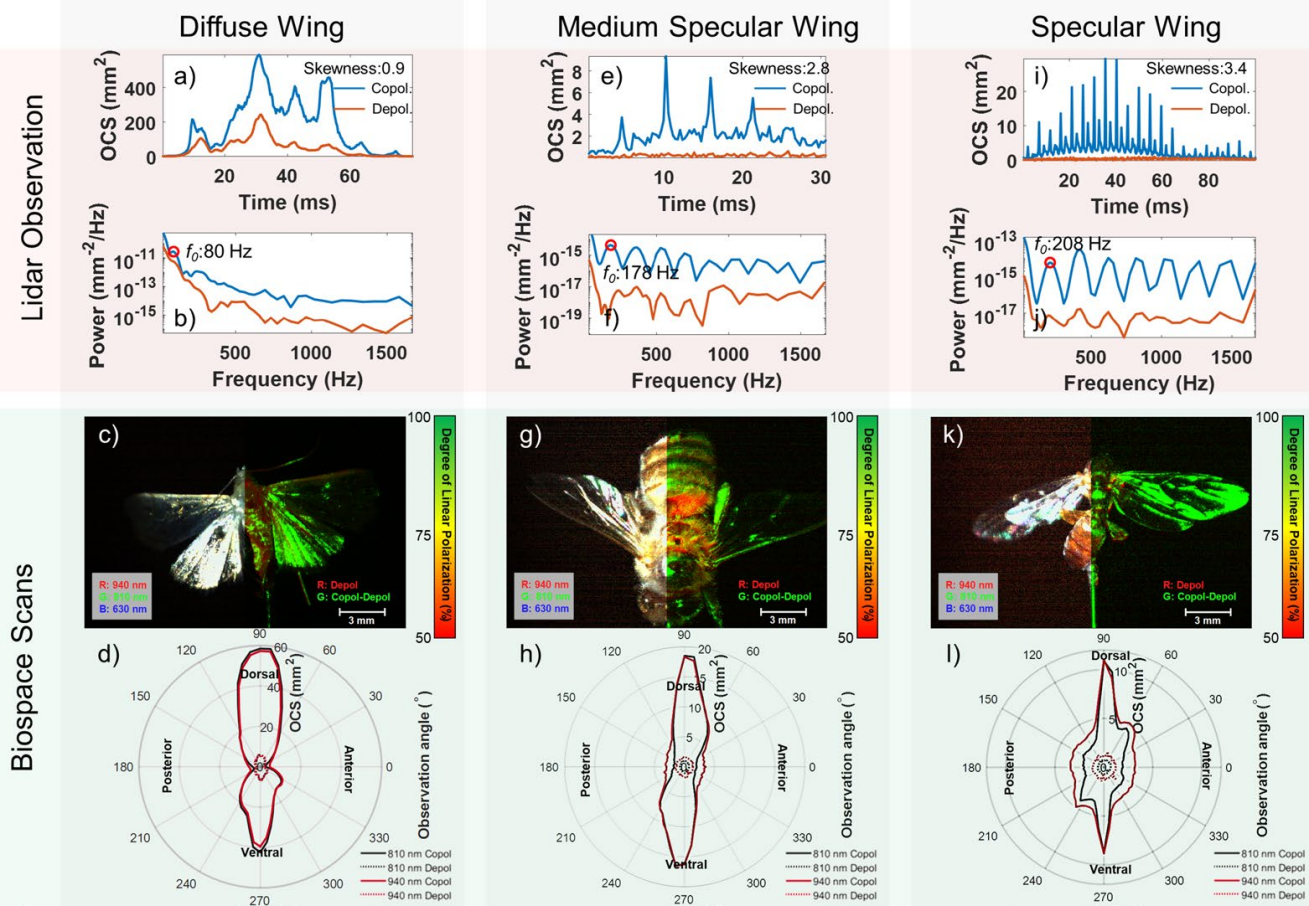
A comparative representation of insect optical property data from two interconnected systems, in-flight insect Lidar observation, and goniometric insect specimen scan. There are three columns displaying a diffuse, a medium specular and a specular wing. Each column is divided into an upper and lower section consisting of each two panels. The upper section provides a display of Lidar-derived insect signals while the lower section, showcases the spectral angular and polarization of selected specimen. Specifically, (**a**) present the time series of a diffuse Lidar observation with (**b**) displaying the corresponding power spectra. (**c**) depict the near-infrared and polarization color image of a captured *Lepidoptera*, the wings are white in color and display a copolarized signal compared to the body. (**d**) showcases the angular reflection at different angles for the diffuse wing of the *Lepidoptera*, with an optical cross section of 60 mm^2^ and a wide angular lobe. Panels (**e**–**l**) show corresponding information for medium specular, and specular wings, and captured *Apis,* and *Diptera* specimen. It is important to note that the Lidar observation and the BIOSPACE scans are not depicting the same insect, but instead insects with similar characteristics.

The retrieved Lidar signal characteristics correlate with the degree of insect wing specularity^39,45,46^. Specifically, Fig. 4 provides a comparative optical characterization of insect signals from two complementary systems: the Lidar system used for in-flight insect observation and the goniometric measurements performed on captured insect specimens. Three representative insect wing types (diffuse, moderately specular, and highly specular) are presented, each with their corresponding Lidar signals, angular reflectance patterns, polarization, and power spectra. Panels are structured to facilitate direct comparison between Lidar observations and controlled specimen measurements, thereby illustrating how insect wing specularity influences observed Lidar signal characteristics. Importantly, these specimens are not the specific individuals detected by the Lidar but they demonstrate the effect of wing specularity on Lidar signals. To determine the relationship between physical traits, angle, and signal, the specimens differing in wing specularity were quantified through angular reflectance measurements obtained using a separate goniometric instrument, BIOSPACE (Biophotonic, Imaging, Optical, Spectral, Polarimetric, Angular Compact Equipment)^47^. Whereas diffuse wings produce broader peaks (Fig. 4a) compared to sharper peaks observed with moderate (Fig. 4e) and high specularity (Fig. 4i)^48,49^. Corresponding differences are observed in the power spectra, with higher specularity leading to more pronounced overtones (Fig. 4c,g,k). Sharper scatter lobes in the polar plots (Fig. 4d,h,l) indicate increasing specularity.

In addition to specularity, the degree of linear polarization on insect bodies and wings is an optical property that can be used to classify insects^46,50^. Both BIOSPACE and the Lidar measure this degree of polarization, illustrated across all panels in Fig. 4 for the three BIOSPACE-scanned insects and the Lidar observations. Finally, Lidar time series modulation content including wingbeat frequencies are well-established parameters for insect classification^38^. The Lidar’s high sampling rate facilitates the detection of insects’ wingbeat frequencies, and the variation in wingbeat frequency is illustrated in Fig. 4b,f,j, representing 80 Hz, 178 Hz, and 208 Hz, respectively.

### Insect diversity estimation with Hierarchical clustering analysis (HCA)

**Fig. 5:**
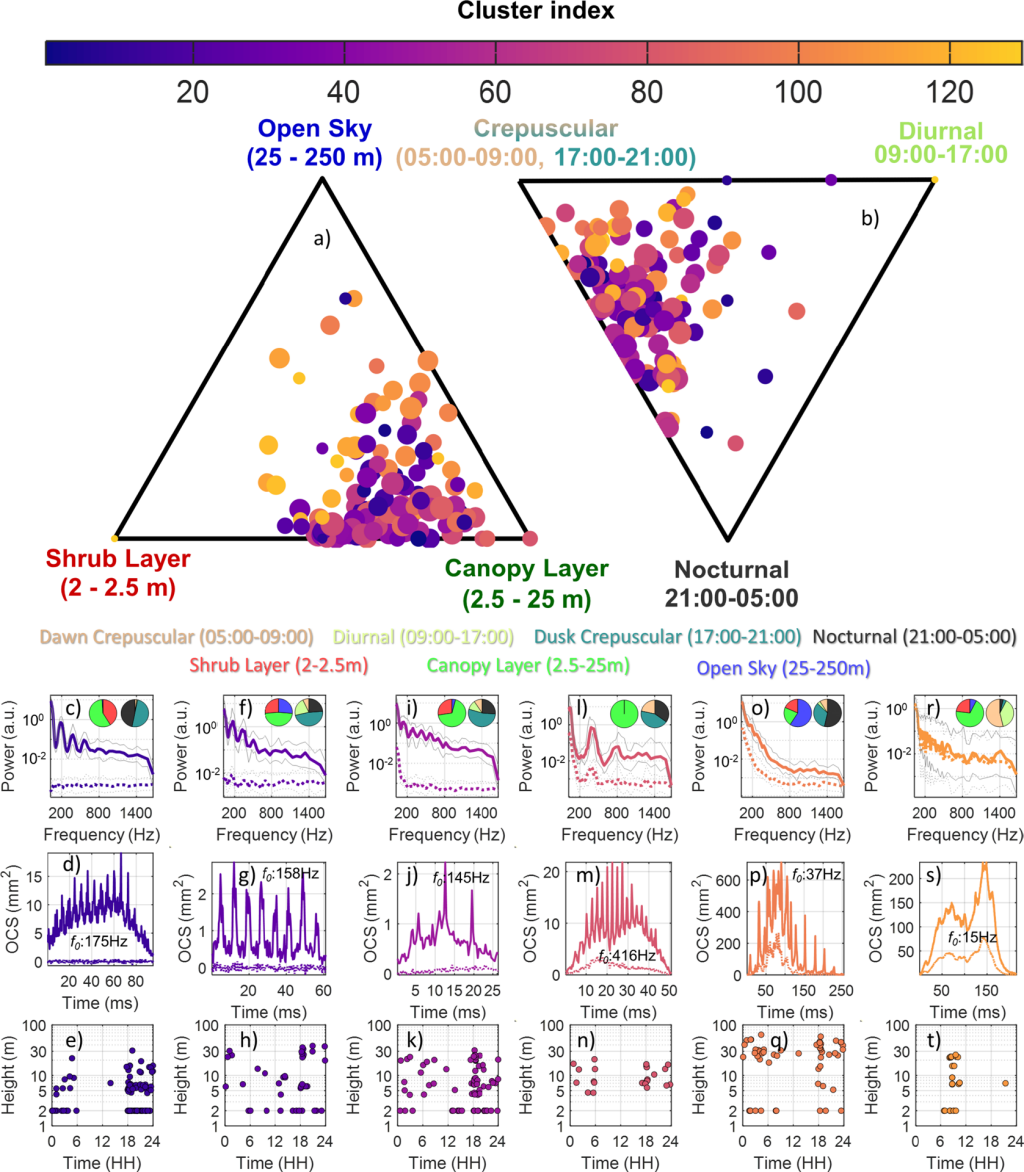
The spatial and temporal distributions of 129 clusters from Lidar observations, with 6 specific cases selected for in-depth examination. (**a**) The ternary plot depicts the 129 clusters’ ratios of distribution across the tree layers. (**b**) Temporal distribution of cluster activity illustrating the main activity times for the cluster dawn and dusk (crepuscular), diurnal, and nocturnal. The scatter dot sizes within these plots are proportional to each cluster’s observations’ square root. The cluster number is indicated by the color of the dots. Dot placement reflects cluster activity distribution across vertical layers and temporal activity patterns. Color coding distinguishes distinct clustering groups. Proximity to a layer corner (e.g., ‘Canopy Layer’) indicates cluster activity predominantly in that specific layer. Similarly, proximity to a temporal corner (e.g., ‘Nocturnal’) imply peak cluster activity during that period. Six clusters were selected to be representative of different insect groups. The selected clusters’ modulation spectra and polarization are detailed in panels (**c**,**f**,**i**,**l**,**o**,**r**) with logarithmic frequency bins. The distribution of clusters is shown in pie charts, with the left charts displaying frequencies across different times (shrub layer: red, canopy layer: green, open sky layer: blue) and the right charts showing frequencies across tree height intervals (dawn: beige, diurnal: bright yellow, dusk: teal, nocturnal: black). Panels (**d**,**g**,**j**,**m**,**p**,**s**) present representative time series (the time series data is not averaged; the WBF in the figure was calculated directly from the displayed time series) Lidar signals for each of the highlighted clusters, while panels (**e**,**h**,**k**,**n**,**q**,**t**) map out their spatial height and temporal distribution across a three-day measurement period.

Hierarchical clustering analysis (HCA) was used on the modulation power spectra from the 6,962 insect observations inspired by previous studies^34,39,44,51^, see details in the method section. HCA effectively manages the variability of entomological Lidar data by grouping modulation spectra based on their pair-wise similarities. The HCA identified 129 distinct clusters which varied in cluster sizes, with the largest cluster containing 153 observations. By plotting the 129 clusters in ternary diagrams reflecting spatial layers (shrub, canopy, open sky; Fig. 5a) and temporal intervals (crepuscular, diurnal, nocturnal; Fig. 5b), we can observe distinct distribution patterns. Because of the large number of clusters, we highlight six representative examples that exhibit clearly different modulation spectra and wingbeat characteristics, showcasing how HCA can reveal taxonomically or behaviorally relevant grouping patterns in Lidar signals (Fig. 5c–t), while the remainder are available as supplementary figures (S4-S6). In Fig. 5a,b, dot position indicates relative proportional representation, with dot size corresponding to the cluster’s observation count. For each group the average power spectra (Fig. 5c,f,i,l,o,r), a time series example (Fig. 5d,g,j,m,p,s), and scatterplots depicting observation times and heights (Fig. 5e,h,k,n,q,t) are shown. The number of Lidar clusters retrieved has been demonstrated to be correlated with observed insect diversity^39,52^, even if correlating clusters to specific species is not possible. Insects are known to have a ~ 25% within-species variation in wingbeat frequency^53^, due to variations in, *e.g.*, body sizes and flight velocities of insects. The inter-species variation was taken into consideration when choosing logarithmically spaced frequency bins.

Insect diversity from traps is reported to correlate with the number of clusters of unsupervised classification of optical signals^39,54,55^. This correlation is used to investigate how insect diversity varies across microhabitats and the time of day in our study. Our analysis demonstrates the potential to group insects based on Lidar signal characteristics, even when direct species identification is not feasible. However, classification into broader taxonomic groups can be made based on extensive studies of group-specific characteristics^35,39,55^. The examples in Fig. 5c–k suggest a predominance of species belonging to the order Diptera, supported by distinct wingbeat frequencies, specular wing signals, and significant linear polarization. Certain clusters have distinct characteristics that allow for further taxonomic inferences. For instance, Fig. 5l–n likely represents mosquitoes based on their high wingbeat frequencies and their crepuscular and nocturnal activity. Observations in Fig. 5o–q could represent species belonging to the Odonata order based on the low wingbeat frequency, specular wing signals, and them primarily being observed in the open sky. Figure 5r–t might represent butterflies (Lepidoptera), as suggested by their low wingbeat frequency, high wing depolarization signals and diurnal activity.

It is important to note that clusters may contain multiple species with similar characteristics. The putative mosquito cluster in Fig. 5l exhibits predominantly crepuscular and nocturnal activity, located at the canopy level (see inset pie chart). Corresponding information for all 129 clusters is provided in the supplementary material.

**Fig. 6:**
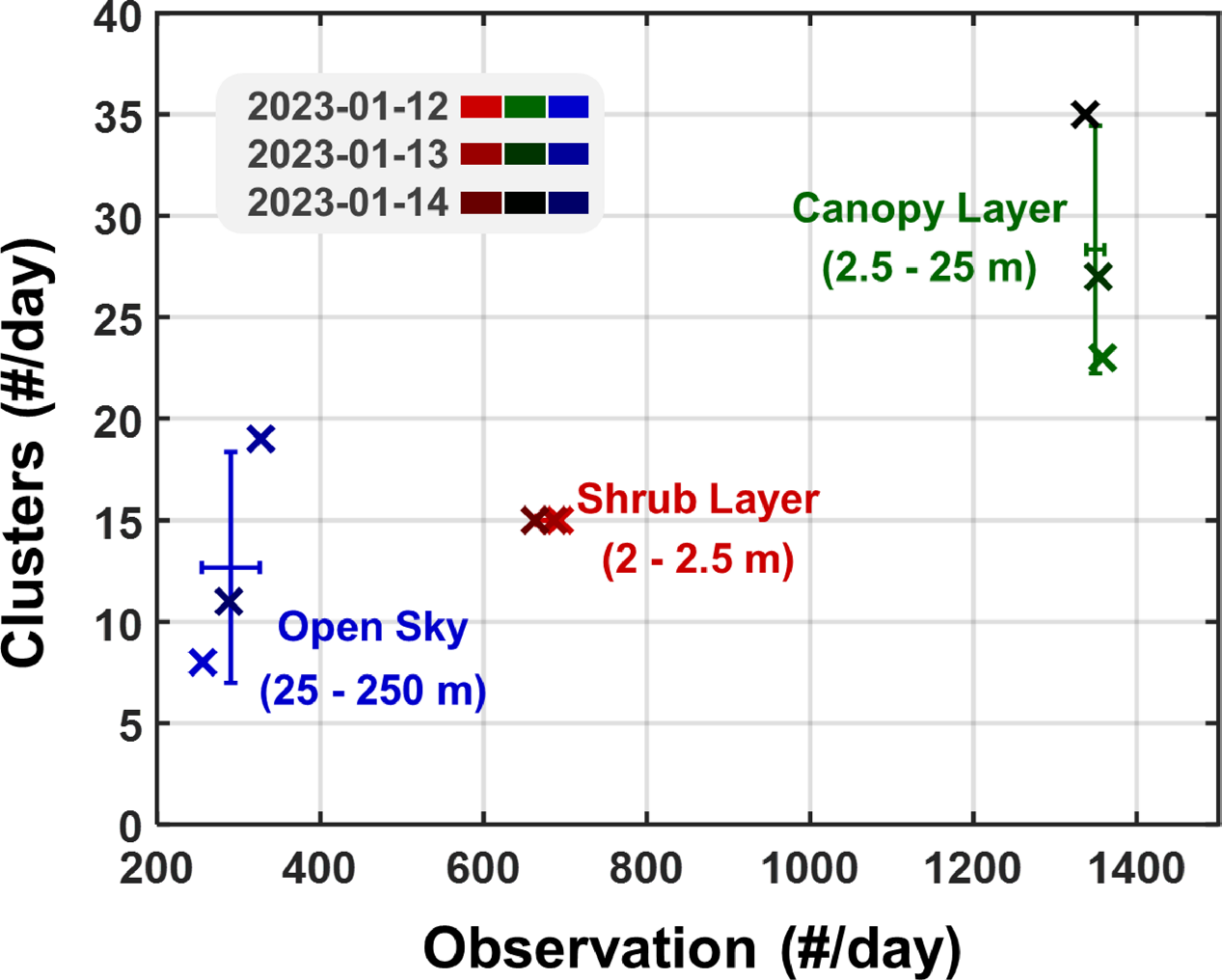
Biodiversity estimates for distinct tree height intervals (Shrub Layer, and Canopy Layer, Open Sky). Consistency is depicted across a three-day measurement period. Error bars are derived from mean and standard deviation calculations. The datapoints are color-coded with the hue representing the layer and the brightness representing measurement date. Note that the system did not allocate equal scanning time across layers and that detection range also differs.

Throughout the three-day study, the number of observations within the shrub, canopy, and open sky layer remained relatively consistent (Fig. 6). However, the number of clusters per layer varied more strongly, indicating that this metric is not simply scaling with insect abundance. Further, it is reported that cluster counts can reflect insect biodiversity^54,55^. The canopy layer consistently had the highest number of observations and clusters throughout the study, highlighting the importance of height-specific insect trapping for estimating diversity, as previously reported^56^. We note that the system did not allocate equal scanning time across the shrub-, canopy-, and sky layers, and the detection range and probe volume was not equivalent. The scrub layer was sampled with a smaller volume, while sky observations started and extended to greater distances (see Fig. 2), resulting in the cluster counts for each layer not directly comparable. Nevertheless, the consistent trends observed over the three-day period within the same region suggest that the relative comparisons between layers remain meaningful for assessing comparative diversity.

## Discussion

Our study investigates the spatial distribution and activity patterns of insects across vegetation layers, highlighting substantial variation in activity and species composition at different heights and times of day^57^. While prior entomological Lidar studies have achieved similar spatial resolution^30,34,40,41,58^, and horizontal scanning^59^ our approach introduces vertical scanning of the Lidar transect at multiple elevation angles, enabling the disentangling of canopy height from the observation range. This advancement addresses range biases and mitigates microhabitat influences on insect monitoring.

We show that Lidar observations outnumber those obtained by conventional trapping methods, while simultaneously being less labor-intensive and less prone to biases^16,17,60^. Its ability to provide high-resolution temporal and spatial data gave us a detailed insight into insect dynamics in ways otherwise unattainable^60^. Based on the Lidar data, we identified distinct activity patterns across the canopy layers (see Fig. 3). The open sky layer had its major peak during the late afternoon, while the shrub and canopy layers both also had significant morning peaks. The activity patterns are greatly influenced by microclimatic conditions (see Fig. 2), with sun exposure, humidity, and temperature differences across the canopy influencing insect activity. For example, higher temperatures coincide with significantly reduced insect activity in the shrub layer (see temperature curves from the shrub and canopy layers in Fig. 3b–d). Rainforests, with intricate canopy stratification, are reported to host different insect species in the different canopy layers, as these layers provide divergent niches^61^. Further, the time of day is reported to affect the insect abundance and diversity, although to a lesser degree^56^. Our findings add to this evidence, as we find a variation in abundance, clusters, and daily activity patterns at different microhabitats in the rainforest canopy, indicating the importance of high spatial and temporal resolution to resolve the full scope of insect diversity in complex rainforest canopies.

Our manual trapping methods, malaise traps, sweep nets, and zipline traps, proved to be labor-intensive and each came with its limitations. Malaise traps, while effective, were found to miss capturing certain species like grasshoppers and dragonflies. Sweep nets were predominantly effective for ground-level captures, and zipline traps, designed to mimic the Lidar beam path, had speed constraints that affected their overall capture rates. Traps have, in addition to the aforementioned biases, practical limitations on placements affecting not only the sampling resolution in time and space but also the locations probed^16^. Typically, the shrub layer is more convenient to measure with traps compared to the inaccessible canopy and open sky layers. This inaccessibility results in additional microhabitat bias since, as we show, biodiversity can vary significantly between different layers. A significant limitation of the traps used in this study was our inability to extend insect trap catch identification to the species level due to constraints in appropriate conditions for preserving insects. Additionally, on-site classification was unfeasible due to limited resources, time, and taxonomists trained on local insect fauna.

A limitation of entomological Lidar is the current inability to identify insects at the species level. An approach towards closing this gap between Lidar observations and species identification is explored by complementing Lidar observations with optical characterization of insect trap catches with BIOSPACE^47^. We documented both scatter phase functions for wings of different reflectivity types and polarimetric goniometric optical information for specimens. This sheds light on the specific dynamics of three distinct insect wing and body characteristics: wing-body specularity, wing-body depolarization ratios, and wing-body melanization (through intensity ratios at different wavelength bands). By relating BIOSPACE-measured insect features to Lidar signals, we gain insights that enhance our ability to interpret Lidar observations in terms of known insect characteristics. Combining these features with established wingbeat frequency data^38^ refines the characterization of insect Lidar observations^35,39^ and allows for comparison to a BIOSPACE-derived library. This comparison can then be used in future research to link individual Lidar observations to specific BIOSPACE-characterized species.

HCA of Lidar-derived power spectra provided deeper insights into insect diversity and spatial distribution across canopy layers. The analysis revealed consistent patterns in cluster richness, with the canopy layer exhibiting the highest diversity. This observation aligns with the ecological complexity and niche diversity typically associated with canopy habitats^61^. By clustering Lidar signal with noise controls, we minimized biases and reliably differentiated true insect clusters from background noise^39^, reflecting ecological variations across height and time of day. The ability to resolve clusters with high temporal and spatial resolution highlights the potential of entomological Lidar for detecting nuanced patterns in biodiversity. Further refinement of clustering methods, particularly through the integration of labeled datasets, could enable, *e.g.*, machine learning techniques, to achieve precise insect characterizations in future studies. Such datasets could be developed using Lidar observations of known species or BIOSPACE measurements of captured insects, as explored in this study. While a one-to-one correspondence between the number of clusters and species richness is not expected, a diverse ensemble of species would also result in a diverse ensemble of signals. In a prior study^51^, photonic insect signal diversity correlated to 70% to Malaise trap based diversity of insect families. By testing synthetic compositions of observations from cages with single species, some 30–40 species could be differentiated with that system. Similarly, we can expect that our current Lidar have a limit for differentiable species much below the present number of species in the Taï forest. Additional bands could improve specificity however.

In conclusion, our study offers insights into insect temporal activity patterns, spatial distribution, and vertical stratification in forest canopies. It demonstrates how Lidar technology can overcome many traditional monitoring challenges, reducing biases and offering a comprehensive view of insect diversity and its variations within microhabitats and over the time of day. However, considering the 12% operational duty cycle of the Lidar within this study and the brief three-day January observation window, our current findings only offer a glimpse into insect activity. The limited number of observations can constitute a bottleneck for diversity estimation. Higher duty cycles and longer ranges of the same system demonstrated a 100-fold increase of observation numbers^62^.The findings of consistent and measurable differences in insect abundance, composition, and temporal activity on small spatial scales and with factors as subtle as shade regime have implications for how to correctly gauge insect biodiversity at a larger scale. Such measurements are relevant for the conservation of insects in the face of the threat of mass extinction. In addition, the non-invasive nature of Lidar data collection provides an ethical approach to studying sensitive populations or protected areas. To achieve a comprehensive understanding of species discrimination and seasonal patterns, extended, multi-seasonal data collection is essential. We conclude that entomological Lidar is an effective tool for insect monitoring with high spatial and temporal resolution with accessibility to locations that are challenging to monitor with conventional traps. This suggests potential for expanding the horizons for entomological Lidar in diverse habitats beyond forests, including plantations, farms, and urban habitats.

## Materials and methods

### Lidar system and scanning procedure

The Lidar system was deployed at a forest edge in the Taï rainforest (5°49′59.5"N 7°20′32.8"W). The transmitter and receiver telescopes were positioned on an 814 mm baseline, supported by a tripod (EQ8, SkyWatcher, China). The Lidar used the Scheimpflug configuration with an angled transmitter, receiving lens, and sensor to measure high-resolution ranging up to 500 m.

The transmitter telescope expanded two 3W TE-polarized 808 nm laser diodes. The polarization of one of the laser emission was rotated by 90° using a wide-angle polymer half-wave plate and was combined with the other laser beam using a polarization beam combiner. The transmitter telescope had a diameter of Ø 75 mm and a focal length of 300 mm. The receiver module was a Ø150 mm diameter and an f600 mm focal Newton telescope (TeleskopService, Germany). A CMOS line sensor (OctoPlus, Teledyne e2v, USA) comprising 2048 pixels, each with dimensions of 10 × 200 μm^2^ was placed in the focal plane of the Newton telescope. According to the Scheimpflug condition and the hinge rule, the CMOS sensor was tilted at a 37° angle relative to the optical axis of the Newton telescope.

The system operated at a 10 kHz sampling rate, with a 100 µs line rate and 80 µs exposure time. Each data file, capturing 3 s of observations, contained approximately 30,000 exposure lines, with an average file size of around 120 MB. A custom LabVIEW script was employed for continuous Lidar data acquisition and file organization.

Over a two-week period, various measurements were conducted in the rainforest. The data analyzed in this paper originates from a continuous 3-day Lidar measurement. During these 3 days, a total of 6 terabytes of raw data was collected. However, after filtering for relevant observations, the dataset was reduced to 180 gigabytes, 3% of its original size.

The Lidar system was programmed to periodically adjust its elevation angles, cycling through 0, 3, 6, 10, 13, 17, and 20°. At 0°, the beam terminated at the board covered with neoprene positioned approximately 80 m away. At 3°, 6°, and 10°, it targeted different canopy layers. At 13°, 17°, and 20°, the beam extended beyond the treetops into the sky. Due to the necessary pauses and adjustments for canopy scanning, the Lidar was operational for 12% of the total measurement duration.

### Lidar observation calibration

Conducting experiments in the jungle presented challenges, primarily due to the lack of a direct power supply for the Lidar system. The system was powered by a generator (Champion Inverterelverk 2200 W, Sweden), which required periodic refueling (~ 7 min, 4 times a day), resulting in temporary gaps in data collection when the generator was powered off for refueling. The bias in the data collection introduced by refueling breaks was however correctable. Importantly, these biases did not significantly impact the overall conclusions, as they were effectively addressed during data analysis and interpretation. The compensation method for observation hours was demonstrated in a previous study^58^.

### Signal diversity estimation

Observational data revealed a consistent trend in insect distribution versus transit time across all three segmented tree heights on different dates (see Fig. S1 a-d). Longer transit time observations were more prevalent in the canopy and shrub layers. To analyze the species diversity at different heights and days, we implemented a HCA on the power spectra derived from Lidar signals and environmental noises, inspired by previous studies observations inspired by previous studies^34,38,39,44,51^. The analysis was initiated with a linkage procedure to calculate cluster distances, based on the pairwise similarity of observations. To minimize potential biases, we processed instrument noise through the same data pipeline as used for the Lidar signals, functioning as a negative control. The following equations and methods were originally developed in another Lidar study with observations from multiple habitats^62^. To assess insect diversity on different days and at different layers, we first computed the pairwise statistical distance, *D(a,b)*, between all power spectra, reflecting their similarity:

1 $$D\left( {a,b} \right) = \sqrt[2]{{\mathop \sum \limits_{{f = 40\;{\text{Hz}}}}^{{1666\;{\text{Hz}}}} \left( {\log \left( {\frac{{P\left( {f,a} \right)}}{{\mathop \sum \nolimits_{{40\;{\text{Hz}}}}^{{1666\;{\text{Hz}}}} P\left( {f,a} \right)}}} \right) - \log \left( {\frac{{P\left( {f,b} \right)}}{{\mathop \sum \nolimits_{{40\;{\text{Hz}}}}^{{1666\;{\text{Hz}}}} P\left( {f,b} \right)}}} \right)} \right)^{2} }}$$

where *a,b* ∈ * {1,…,N}* and *a ≠ b*, are two observation indices, *N* is the number of observations. *P(f,a)* denotes the modulation power at frequency *f* for observation *a*. To ensure we compare only the shapes of these spectra, all power spectra were normalized by their respective total power, and the powers were log-transformed before computing the Euclidean distance. This approach makes the distance metric sensitive to harmonic structure rather than overall magnitude (which can vary due to differences in range and beam-insect overlap). The resulting distances were then hierarchically clustered into a linkage tree, *Z*_*(p)*_, where *p * ∈ *{1,…,N − 1}* represents pairs. This tree compresses the pairwise distance matrix by sequentially merging the most similar spectra. To control for instrument noise and environmental artifacts, we repeated this procedure using noise fragments, yielding a corresponding linkage tree *Z*_*ξ(p)*_. Both *Z*_*(p)*_ (insect data) and *Z*_*ξ(p)*_ (noise control) exhibit a general decline in linkage values when sorted in descending order (see Supplementary Figure S1). To detrend linkages, calculated compensated linkages, *Z*_*comp.(p)*_, by multiplying the median slope accordingly:

2 $$\begin{array}{*{20}c} {Z_{comp.\left( p \right)} = \left( {\frac{N - 1}{p}} \right)^{\beta } Z_{\left( p \right)} } & {,\beta = \left| {\frac{{\Delta \log Z_{\left( p \right)} }}{\Delta \log p}} \right|_{median} } & {p \in \left\{ {1 \ldots N - 1} \right\}} \\ \end{array}$$

where *β* is the median slope. Finally, we determined the number of clusters, *NoC*, by counting how many compensated linkages exceed the median plus the interquartile range (*IQR*) of those same compensated linkage values (outlier criteria):

3 $$NoC = \mathop \sum \limits_{p}^{N - 1} \left[ {Z_{comp.\left( p \right)} > \left( {\left| {Z_{comp.\left( p \right)} } \right|_{median} + \left| {Z_{comp.\left( p \right)} } \right|_{IQR} } \right)} \right]$$

We identified *NoC* equivalently for noise fragments and found only a couple for linkages exceeding this criterion (see Supplementary Fig. S7). As in a previous study^62^, we consider *NoC* to represent the number of distinguishable power spectra from noise. For the clustering in Fig. 5 a single HCA was applied to all observations across days and layers. For Fig. 6, HCAs were applied separately and *NoC* was evaluated independently between days and layers illustrating the consistency of the estimate.

### Conventional trapping configuration

To manually sample the insect fauna at different locations and canopy heights, various types of traps were utilized, each selected based on their advantages and limitations^63^. The traps were emptied and cleaned at specified times of the day.

#### Malaise traps

Two Malaise traps were employed at different altitudes—ground level (~ 1 m) and canopy top (~ 20 m). These traps were emptied four times a day, specifically at 5 am, 10 am, 5 pm, and 10 pm. These timings were selected to maximize insect capture before and after their peak activity periods (dawn, dusk crepuscular). The Malaise trap, typically exhibiting the least bias compared to bait and light traps^16^, aimed to capture a diverse distribution of fauna at both the ground and canopy levels. However, larger insects such as grasshoppers and dragonflies were often not captured despite being frequently observed on site. We used dry collection bottles, potentially enabling some insect groups to escape.

#### Zipline traps

Zipline traps were utilized and emptied four times a day: at 6:30 am, 7:30 am, 5:30 pm, and 6:30 pm. The primary objective of the zipline trap was to capture insects that followed a path similar to the Lidar beam, representing the insects observed in the Lidar signal. However, due to the system’s slow movement (about 1 m/s travel speed), many in-flight insects often evaded the net. This limitation indicated the potential benefit of a motorized zipline system with a faster speed, around 10 m/s for increased catches.

#### Active sweep netting

Parallel to the zip line trapping, active sweep netting was conducted four times a day, at 6:30 am, 7:30 am, 5:30 pm, and 6:30 pm. This method was executed with care, sweeping only just above the grass level to try to avoid provoking grass-dwelling insects into jumping into the net. This precaution was taken to prevent the potential overrepresentation of these species in the data. The aim was to ensure that the insects captured were in flight during collection. The selected times ensured adequate sunlight for safe movement, given the jungle’s lack of electricity, while also targeting insects before and after dusk and dawn.

### Insect handling, documenting and selection

**Insect Documenting:** Insects were euthanized and placed in individual bags labeled with the time, location, and method of capture. Insects were identified to the order level, and their counts and size classes were recorded. Photographs of all catches were taken using both cellphones and microscopes, with a scale marker included for reference.

**Insect Selection for BIOSPACE Study:** Insects that were frequently captured and that fit the size criteria for BIOSPACE imaging, typically those with a wingspan of 0.5 to 1.5 cm, were chosen for further examination. The wings of these selected insects were spread and photographed using higher-quality cameras. Samples with minimal damage and either diffused or clear wings were specifically selected for scanning. For beetles, their elytra (wing covers) were carefully opened, and their wings were spread to enhance wing signal acquisition. Insect specimens were identified to the order level for general taxonomic categorization.

**Scanning with BIOSPACE:** The selected insects were mounted in BIOSPACE^47^ and scanned with different rotation angles. For each angle, images with different spectral (eight wavelength bands from 365 to 940 nm) and polarization information were captured. The same procedure was conducted with the removed wings of the insects, to measure their specularity. The measurements were conducted on-site with the portable BIOSPACE, placed in a dark box to minimize the ambient signal.

## Electronic supplementary material

Below is the link to the electronic supplementary material.

Supplementary Material 1

Supplementary Material 2

## Data Availability

All data are available in the main text or the supplementary materials.
