# Supplementary material for: Stratification of insect diversity and daily activity patterns in the West African virgin forest Taï assessed by entomological Lidar
Source: Sci Rep. 2025 Jul 15;15:25663. doi: 10.1038/s41598-025-05200-z (PMC12263827; doi:10.1038/s41598-025-05200-z)
Supplement: Supplementary file 2 — Supplementary Material 2 [file 41598_2025_5200_MOESM2_ESM.pdf]

Supplementary Materials

Materials and Methods

Figs. S1 to S8

Figures and Tables:

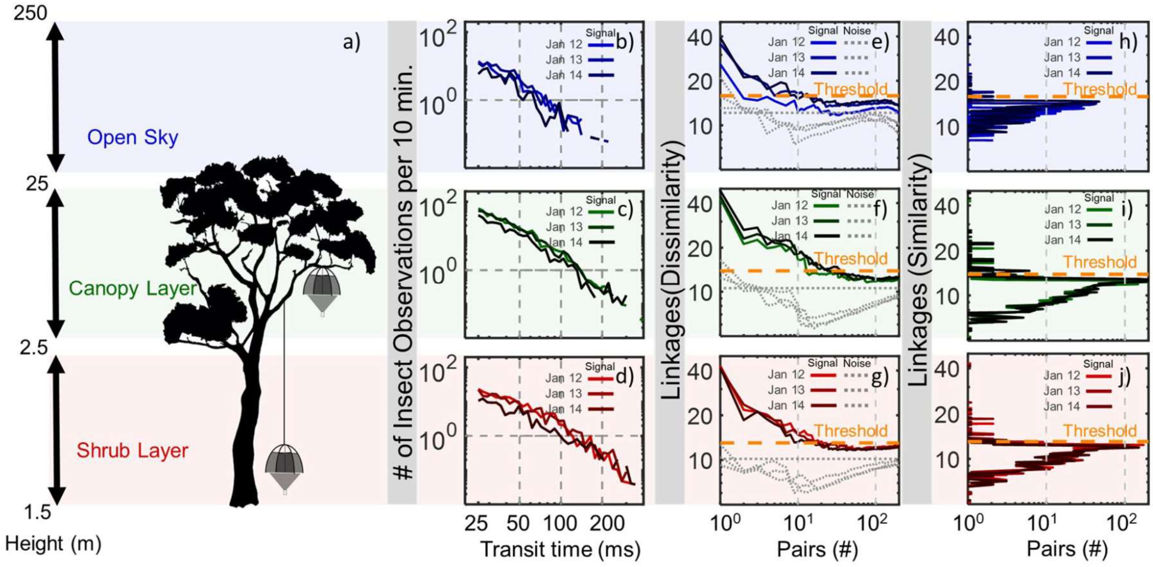

Figure S1: a) A schematic representation details the placement of two Malaise traps on a tree, segmenting tree heights into the shrub layer (0-2.5 m), the canopy layers (2.5 m - 25 m), and the open sky (25 m and above). Note: This depiction is not to scale. b, c, d) Histograms display transit time distributions for observations, differentiated by date and height intervals. e, f, g) Linkages, based on dissimilarity values, are plotted against the number of pairs. h, i, j) Demonstrations of linkages, emphasizing similarity, are related to the number of pairs.

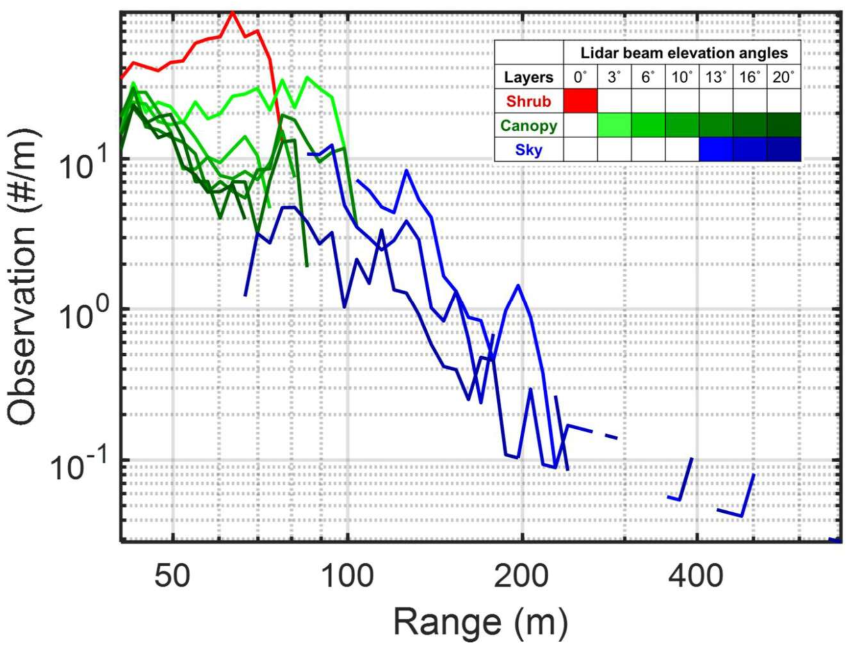

Figure S2: This graph illustrates the observation range in relation to the number of observations for different beam elevation angles.

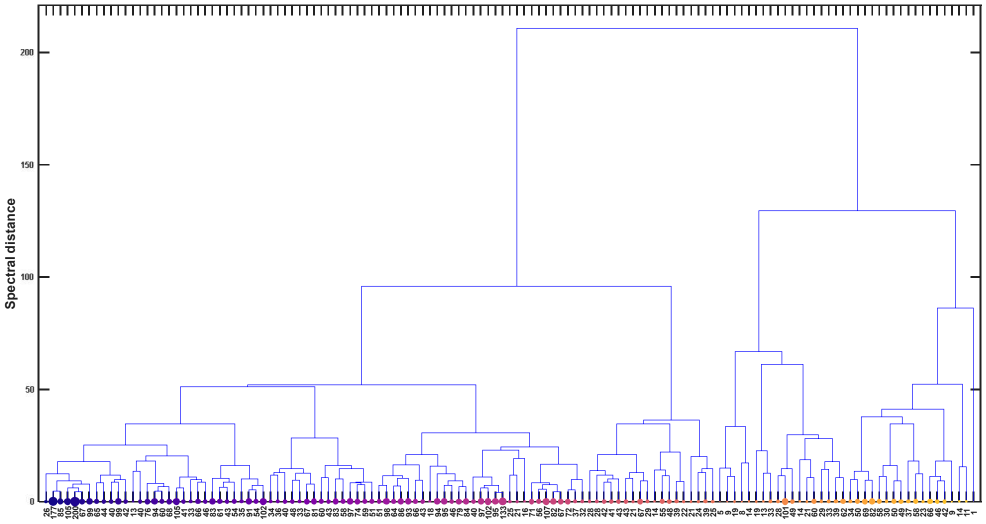

Figure S3: We clustered 6,962 LIDAR observations into 129 unique clustering groups, and this dendrogram illustrates the hierarchical arrangement of these clusters.

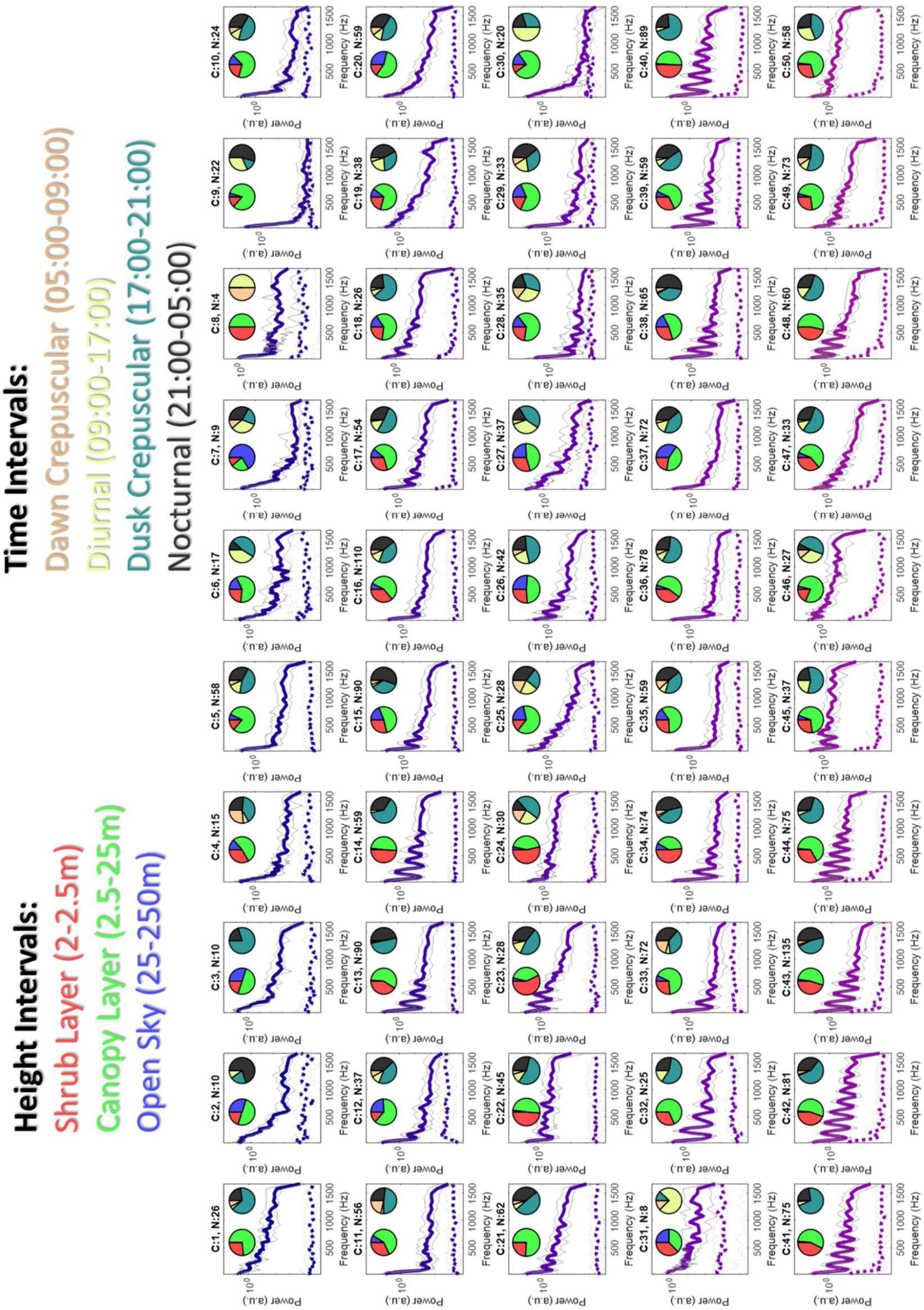

Figure S4: The modulation spectra of clustering groups numbered from 1 to 50 among the 129 clusters. Accompanying pie charts provide insights into the frequency distribution of these clusters across different times of the day and tree height intervals.

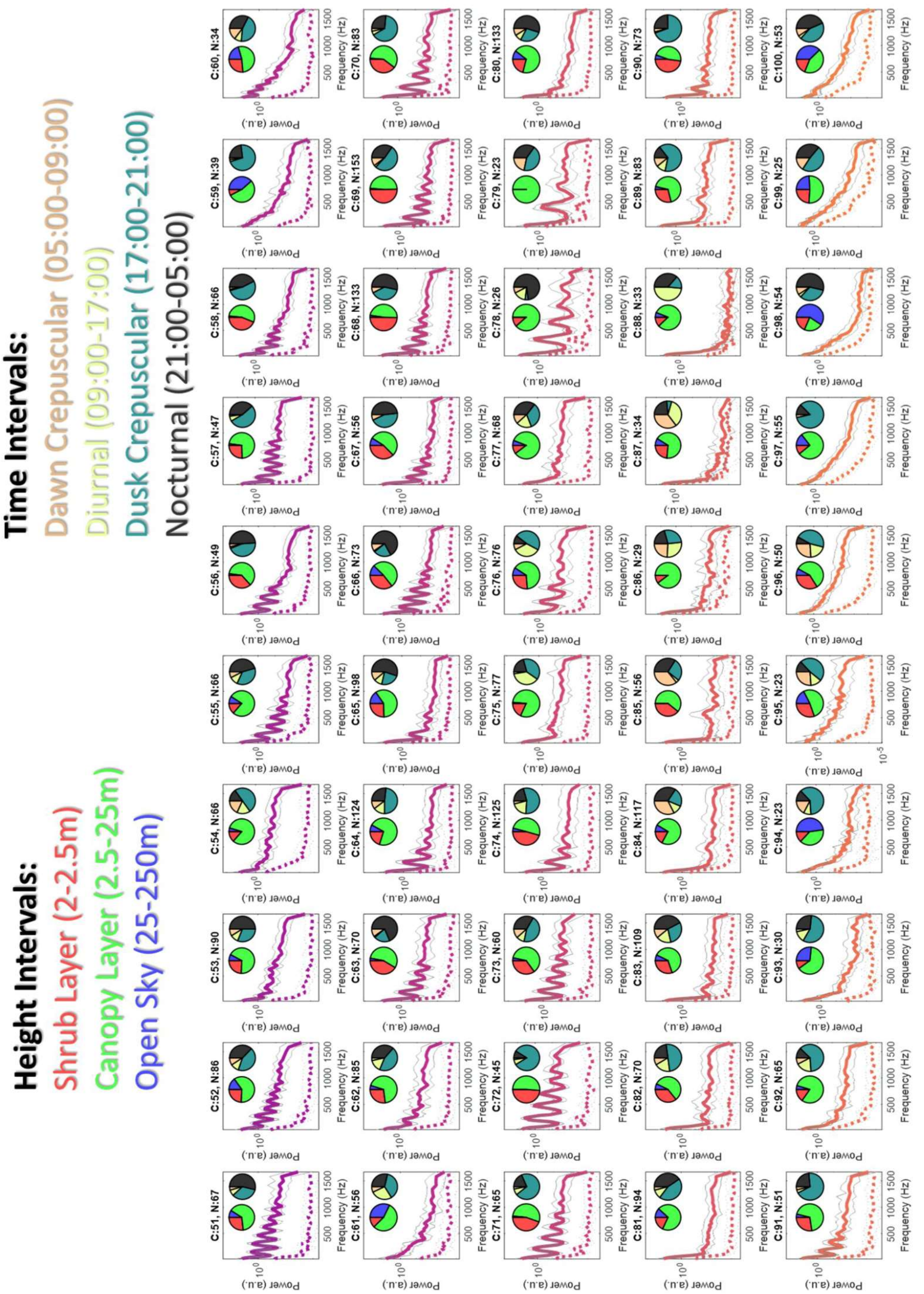

Figure S5: The modulation spectra of clustering groups numbered from 51 to 100 among the 129 clusters. Accompanying pie charts provide insights into the frequency distribution of these clusters across different times of the day and tree height intervals.

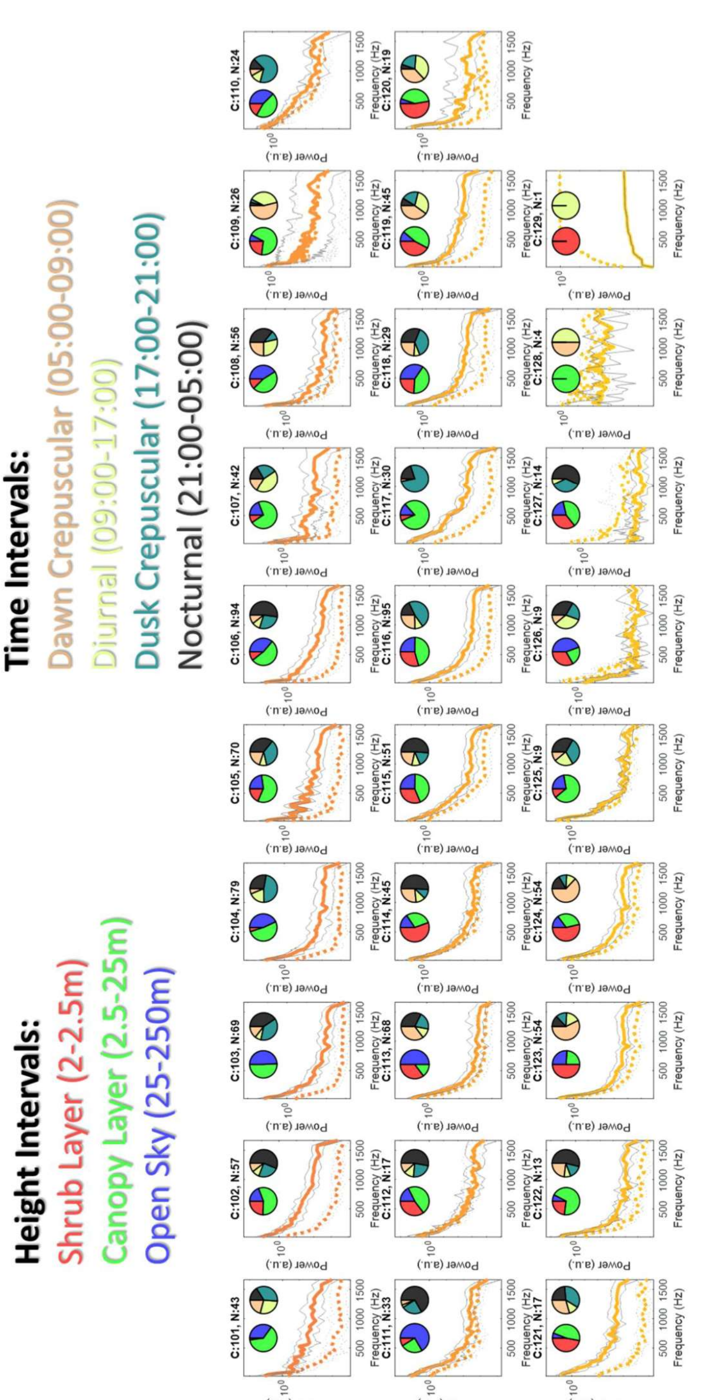

Figure S6: The modulation spectra of clustering groups numbered from 101 to 129 among the 129 clusters. Accompanying pie charts provide insights into the frequency distribution of these clusters across different times of the day and tree height intervals.

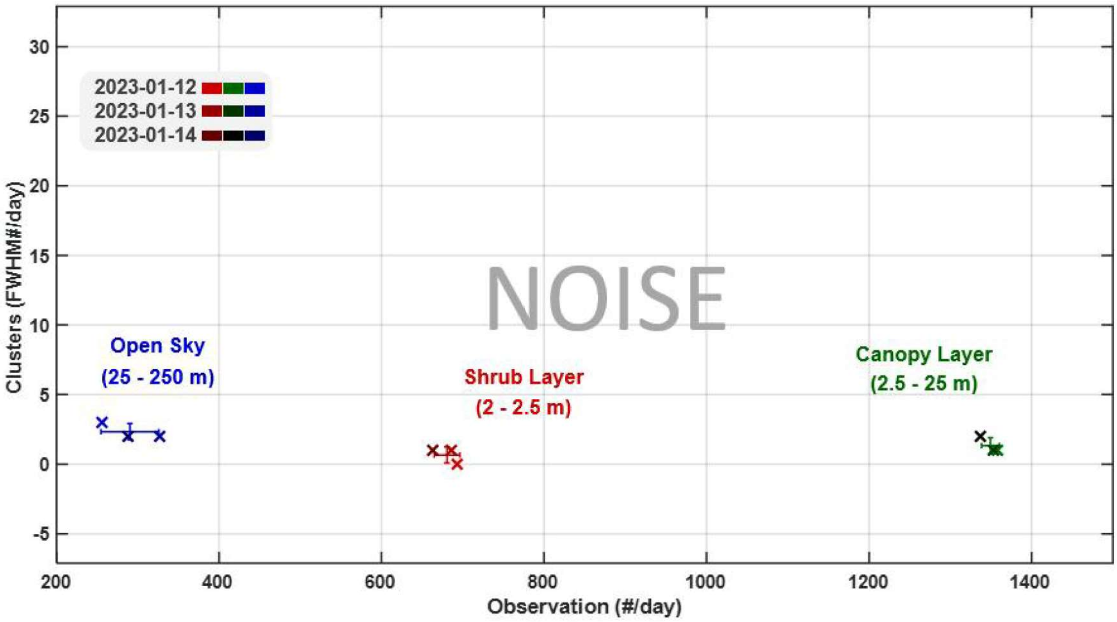

Figure S7: Validation of Biodiversity in Lidar Signals Against Noise - This figure demonstrates the analysis conducted to confirm that the biodiversity detected in the Lidar signals is not due to random noise. Noise levels were clustered within the shrub, canopy, and open sky layers, revealing no significant diversity or difference in clustering between these ecological layers.
